# Gelatin-Modified Calcium/Strontium Hydrogen Phosphates Stimulate Bone Regeneration in Osteoblast/Osteoclast Co-Culture and in Osteoporotic Rat Femur Defects—In Vitro to In Vivo Translation

**DOI:** 10.3390/molecules25215103

**Published:** 2020-11-03

**Authors:** Benjamin Kruppke, Seemun Ray, Volker Alt, Marcus Rohnke, Christine Kern, Marian Kampschulte, Christiane Heinemann, Matthäus Budak, Josephine Adam, Nils Döhner, Lucretia Franz-Forsthoffer, Thaqif El Khassawna, Christian Heiss, Thomas Hanke, Ulrich Thormann

**Affiliations:** 1 Institute of Materials Science, Technische Universität Dresden, Max Bergmann Center of Biomaterials, 01069 Dresden, Germany; Christiane.Heinemann@tu-dresden.de (C.H.); Josephine.Adam@tu-dresden.de (J.A.); Thomas.Hanke@tu-dresden.de (T.H.); 2 Department of Trauma, Campus Giessen, Hand and Reconstructive Surgery, University Hospital Giessen-Marburg GmbH, Rudolf-Buchheim-Str. 7, 35385 Giessen, Germany; seemun.ray7@gmail.com (S.R.); Volker.Alt@chiru.med.uni-giessen.de (V.A.); Matthaeus.Budak@chiru.med.uni-giessen.de (M.B.); Nils.Doehner@med.uni-giessen.de (N.D.); Lucretia.Franz-Forsthoffer@med.uni-giessen.de (L.F.-F.); Thaqif.ElKhassawna@chiru.med.uni-giessen.de (T.E.K.); Christian.Heiss@chiru.med.uni-giessen.de (C.H.); Ulrich.Thormann@chiru.med.uni-giessen.de (U.T.); 3 Experimental Trauma Surgery, Justus-Liebig-University of Giessen, Rudolf-Buchheim-Str. 7, 35385 Giessen, Germany; 4 Institute of Physical Chemistry, Justus-Liebig-Universität Giessen, Heinrich-Buff-Ring 17, 35392 Gießen, Germany; Marcus.Rohnke@phys.chemie.uni-giessen.de (M.R.); Christine.Kern@phys.chemie.uni-giessen.de (C.K.); 5 Justus-Liebig-Universität Giessen, Klinik für Diagnostische und Interventionelle Radiologie, Klinikstr. 33, 35392 Gießen, Germany; Marian.Kampschulte@radiol.med.uni-giessen.de

**Keywords:** biomimetic material, osteoporosis, fracture defect, co-culture, calcium phosphate, strontium phosphate

## Abstract

The development and characterization of biomaterials for bone replacement in case of large defects in preconditioned bone (e.g., osteoporosis) require close cooperation of various disciplines. Of particular interest are effects observed in vitro at the cellular level and their in vivo representation in animal experiments. In the present case, the material-based alteration of the ratio of osteoblasts to osteoclasts in vitro in the context of their co-cultivation was examined and showed equivalence to the material-based stimulation of bone regeneration in a bone defect of osteoporotic rats. Gelatin-modified calcium/strontium phosphates with a Ca:Sr ratio in their precipitation solutions of 5:5 and 3:7 caused a pro-osteogenic reaction on both levels in vitro and in vivo. Stimulation of osteoblasts and inhibition of osteoclast activity were proven during culture on materials with higher strontium content. The same material caused a decrease in osteoclast activity in vitro. In vivo, a positive effect of the material with increased strontium content was observed by immunohistochemistry, e.g., by significantly increased bone volume to tissue volume ratio, increased bone morphogenetic protein-2 (BMP2) expression, and significantly reduced receptor activator of nuclear factor kappa-B ligand (RANKL)/osteoprotegerin (OPG) ratio. In addition, material degradation and bone regeneration were examined after 6 weeks using stage scans with ToF-SIMS and µ-CT imaging. The remaining material in the defects and strontium signals, which originate from areas exceeding the defect area, indicate the incorporation of strontium ions into the surrounding mineralized tissue. Thus, the material inherent properties (release of biologically active ions, solubility and degradability, mechanical strength) directly influenced the cellular reaction in vitro and also bone regeneration in vivo. Based on this, in the future, materials might be synthesized and specifically adapted to patient-specific needs and their bone status.

## 1. Introduction

Osteoporotic bone provides inadequate conditions for fracture healing [1,2]. Both, the disturbed ratio of osteoblasts and osteoclasts (high turnover osteoporosis) or the small population and inactivity of both osteoblasts and osteoclasts (low turnover osteoporosis) lead to a decelerated fracture healing. In case of a low turnover state, as it is present in age-related, senile osteoporosis [3], the amount of bone cells is generally small and the cells show low activity. Thus, stagnation of bone remodeling and loss of bone are a result of a combined depression of osteoblasts and osteoclasts activity [4]. Here, the main task of a supporting bone substitute material is the stimulation of cell populations, in particular, that of bone-forming osteoblasts.

High turnover osteoporosis, also known as postmenopausal osteoporosis, is characterized by large populations of bone cells, which are also active but the ratio between osteoblasts and osteoclasts is shifted towards bone-resorbing cells [5]. In this particular case, a bone substitute material-based treatment of bone fractures of patients suffering from high turn-over osteoporosis shall aim at manipulation of osteoclast-formation and/or -activity to avoid serious complications. A promising way of influencing osteoclasts during the process of bone healing is the incorporation of strontium ions into an implantable bone substitute material. The first indications of a positive effect of strontium ions on bone regeneration were already described in the early 1950s [6]. In the following decades, the promoting effect of strontium on bone-forming osteoblasts and their precursors was the subject of several studies and is now mainly known [7,8,9,10]. In the present case, a degradable bone substitute material of gelatin-modified calcium and strontium phosphates facing the special demands of osteoporotic bone was investigated. Strontium ion release from the material was adjusted to directly stimulate osteoblastogenesis by ion release or indirectly increase the activity of osteoblasts after material resorption by osteoclasts.

The materials in this study are the porous specimen of two mixed phases of calcium/strontium phosphate precipitated in presence of gelatin. The two organically modified mineral phases originate from a series of preliminary in vitro degradation and cell culture studies which, starting from pure organically modified calcium phosphate [11] and strontium phosphate, respectively, showed wide variability in degradability and ion release. The initial ratio of the calcium/strontium ions can be used to vary the mineral morphology, structure, and degradability [12]. Especially, the specimen of phosphate-prestructured gelatin mineralized with mixed solutions of calcium:strontium 5:5 (short PPGC + S 5:5) and specimen of PPGC + S 3:7 turned out to be particularly promising for further investigations [13]. As a brief introduction, PPGC + S 5:5 on the one hand is calcium phosphate, brushite with disturbed morphology and crystal structure by strontium substitution with a measured Ca/Sr-ratio of 63%/37% by mass. PPGC + S 3:7 on the other hand is a strontium phosphate slightly doped with calcium ions showing a measured Ca/Sr-ratio of 10%/90% by mass. With an increasing amount of strontium, a decrease in degradation during incubation in physiological solutions and influence on pH as well as phosphate ion release were measured. PPGC + S 5:5 showed high reactivity in physiological solutions as it performs a dissolution and re-precipitation process, which causes high initial calcium and strontium ion release as well as an increase in mechanical strength with time in consequence of the bioactivity of the material, known as mineral precipitation on the specimen surface. Osteoblasts showed good adhesion, proliferation, and differentiation in mono-culture, while human mononuclear cells (hMc) showed fusion to osteoclasts and resorption activity, proven by connexion 43 and 37 as well as tartrate-resistant acid phosphatase isoenzyme 5b (TRAP) activity [12]. PPGC + S 3:7 is less bioactive and, therefore, only an initial increase in cation concentration was present during incubation and no hardening was observed [13]. The osteoblasts showed good adhesion, proliferation, and increased and faster differentiation.

In order to verify the in vitro knowledge and allow translation of the material development to the application, it is first necessary to investigate the material in a clinically relevant osteoporosis model. Since it is known that osteoporosis in particular affects metaphyseal trabecular bone [14,15], a metaphyseal osteoporotic fracture critical size defect in rats was selected [16]. This defect allows testing of the described bone substitute materials and quantifies stimulation of fracture defect healing while still mimicking the clinically relevant fracture situation in osteoporotic patients.

The animal model used to study the effects of the material on bone healing in osteoporotic fractures comprises (a) an osteoporotic bone status of the rats, (b) a metaphyseal fracture, and (c) a critical size defect.

Published models on metaphyseal fracture healing in rat tibiae in which osteotomy with a small gap size of 0.5 mm was performed to analyze systemic anti-osteoporotic treatment [17,18] provide small space for locally applied biomaterials. Furthermore, metaphyseal drilled hole defects [19,20] and partial osteotomy without complete fracture [21] do not consider biomechanical boundary conditions of a defect, which is stabilized on one side with an osteosynthesis plate. Therefore, the previously published rat model with ovariectomy and subsequent calcium-, phosphorus-, vitamin D3-, soy-, and phytoestrogen-free diet was used to induce osteoporotic conditions. Furthermore, a 5 mm wedge-shaped defect in the metaphyseal supracondylar region (critical size defect) with T-plate fixation offers space for implantation and evaluation of bone graft materials.

## 2. Results

For an in-depth biomaterial characterization, first of all, material scientific analyses were carried out, which complemented previously published data and serve to interpret the results of the current study. Subsequently, the results of in vitro cell culture using osteoblast/osteoclast co-culture are presented. Finally, the results of the in vivo biomaterial analysis in the osteoporotic rat model with the metaphyseal femoral defect are shown.

### 2.1. In Vitro Analysis of Material Properties and Cellular Reaction

#### 2.1.1. Morphological Characterization of Calcium/Strontium Hydrogen Phosphates and Specimen

**Figure 1:**
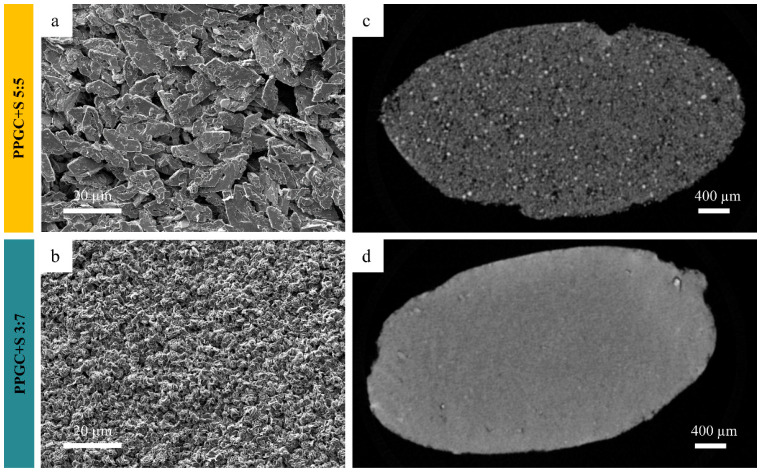
SEM images (**a**,**b**) of just precipitated and dried mineral and µ-CT (**c**,**d**) of the final specimen of phosphate prestructured gelatin mineralized with calcium and strontium ions in the molar ratio of 5:5 and 3:7. µ-CT shows a sectional plane in the base of the specimen (compare Figure 12d).

To assess material degradation after implantation in further chapters, the evaluation of the structure of the test specimens is of great importance. For this purpose, morphological examinations of the mineral phases were first carried out by means of scanning electron microscopy (SEM). Significant differences in the particle morphologies of the mineral phases were present with PPGC + S 5:5 showing large platelet-shaped particles of 5–20 µm dimensions, while PPGC + S 3:7 showed granular particles of only a few µm in diameter (Figure 1a,b). In consequence of the particle morphology, there was also a difference visible in the material density of specimens (Figure 1c,d) in the µ-CT images. It can be seen that the larger and anisotropic mineral particles of PPGC + S 5:5 processed into specimens for implantation caused a higher pore number and size, while the smaller PPGC + S 3:7 particles led to a higher density of the specimen.

#### 2.1.2. Mechanical Strength and Ion Release of Calcium/Strontium Hydrogen Phosphates

**Figure 2:**
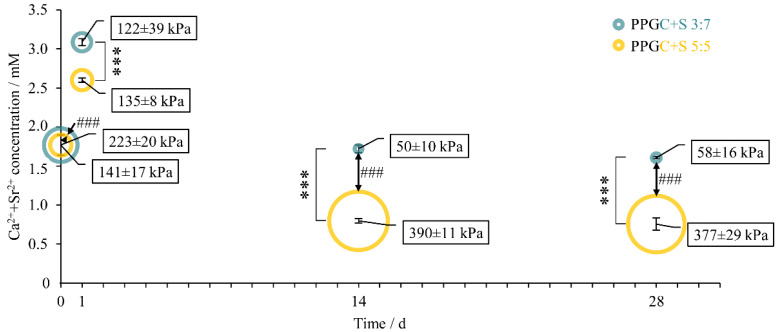
Ion concentrations of calcium and strontium determined colorimetrically over 28 d during static incubation of the specimens in A-minimal essential medium (α-MEM). Bubble size and data labeling indicate the indirect tensile strength of specimens during incubation. Error bars and ± values indicate standard deviation. Significant differences in ion concentrations and indirect tensile strength are indicated by asterisks and hashes, respectively.

Over time, as the samples were stored in a cell culture medium, the cation concentration was first significantly increased in both cases (2.6 mM for PPGC + S 5:5 and 3.1 mM for PPGC + S 3:7; Figure 2) in comparison to the initial concentration of 1.8 mM. Afterward, in the case of PPGC + S 5:5, the concentration was decreased again below the initial concentration on days 14 and 28, whereas PPGC + S 3:7 did not show a significant difference in ion concentration at days 14 and 28 to the initial concentration. The specimen had an initial indirect tensile strength, which differed significantly (bubble size in Figure 2). After incubation for one day, the difference in mechanical strength between PPGC + S 5:5 and PPGC + S 3:7 diminished. In the course of incubation, the specimen of PPGC + S 3:7 showed a continuous decrease in mechanical strength during incubation. In contrast, PPGC + S 5:5 samples showed a significantly increased strength at days 14 and 28.

#### 2.1.3. Osteoblast/Osteoclast Co-Culture

To assess the in vitro influence of the osteoblast/osteoclast ratio, human bone marrow stromal cells (hBMSC) were first pre-cultured on the materials. They showed adherence measured by lactate dehydrogenase (LDH) activity of 54.3 ± 0.4% on PPGC + S 5:5 and 45.9 ± 0.1% on PPGC + S 3:7 (data not shown). The differentiation of hBMSC was induced by the addition of dexamethasone and β-glycerophosphate. Differentiation of hBMSC to osteoblasts was observed with a cell count related alkaline phosphatase (ALP) on day 8 of about 3.1 ± 0.3 nmol/pNP/min/ 10^5^ cells on PPGC + S 5:5, which was significantly exceeded by the cells on the higher strontium containing PPGC + S 3:7 specimen with about 7.7 ± 0.3 nmol/pNP/min / 10^5^ cells (data not shown). These results match with previous investigations focusing on material scientific properties of gelatin modified calcium/strontium phosphates and osteoblasts in mono-culture [12].

**Figure 3:**
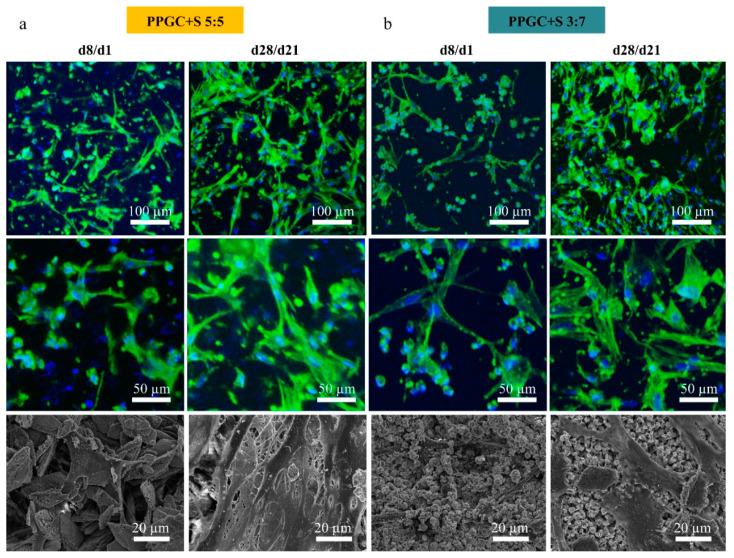
Confocal laser scanning microscope (cLSM) (upper row) and SEM pictures (lower row) of osteoblasts and monocytes/osteoclasts on PPGC + S 5:5 (**a**) and PPGC + S 3:7 (**b**). The actin skeletons (green) and the nuclei (blue) are visualized.

The hMc seeded on the material together with the pre-cultured osteoblasts also showed a good and almost equal adherence of approximately 24.8 ± 0.3% on PPGC + S 5:5 and approximately 22.0 ± 0.1% on PPGC + S 3:7 (data not shown). According to the fluorescence images, the osteoblasts on the material (after 8 days of pre-cultivation) are characterized by an elongated actin skeleton as well as the first contacts of the cells to each other (Figure 3a,b, d8/1). In addition, the adherent hMc can be recognized as mononuclear round cells with less actin between the osteoblasts. At the end of co-culture (after 28 days for osteoblasts and 21 days for osteoclasts), a significant increase in the actin network of osteoblasts was observed on both materials. This was seen in the SEM images, which showed the increase in number and cell spreading of hMSC/osteoblasts on the material. Occasionally, multinucleated, rather round-shaped osteoclast-like cells were seen between the stretched osteoblasts in the confocal laser scanning microscope (cLSM) and on the material surface in the SEM.

For further analysis, the material inherent influences on the osteoblast/osteoclast ratio and their activity were focused on osteoclastic differentiation. Differentiation of monocytes to osteoclasts in co-culture was performed solely by crosstalk with hBMSC since the external addition of differentiation factors such as M-CSF and receptor activator of nuclear factor kappa-B ligand (RANKL) was not used. Furthermore, osteogenic additives were omitted with the start of co-culture to avoid inhibiting osteoclastogenesis [22,23]. Thus, only the material properties influenced the crosstalk of the two cell types.

**Figure 4:**
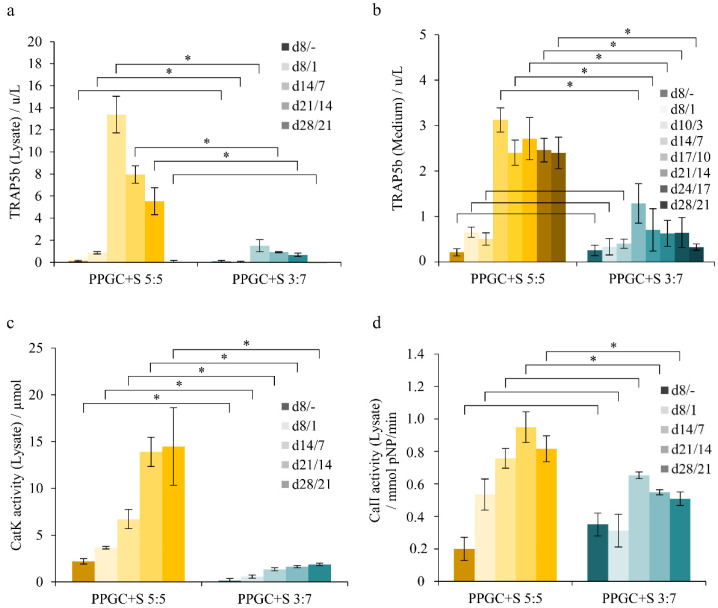
Osteoclastic differentiation of human mononuclear cells (hMc) to osteoclasts during co-cultivation with osteoblasts in vitro. The first number in the legend refers to the duration of the precultivated HMSC and the time required for differentiation to osteoblasts. The second number refers to the time of human monocytes cultivation seeded on day 7 to the osteoblasts, giving the time of co-cultivation. Intracellular tartrate-resistant acid phosphatase isoenzyme 5b (TRAP5b) (**a**) as well as extracellular TRAP5b expressed into the medium (**b**) as an early marker of osteoclastogenesis and cathepsin K (CatK) activity (**c**) and carbonic anhydrase II (CaII) activity (**d**) as an indicator for osteoclastic resorption. Asterisks indicate statistical significant differences (*p* < 0.05).

During co-culture, the inhibitory effect of the high strontium containing PPGC + S 3:7 specimens became obvious. The PPGC + S 3:7 specimen caused a reduced differentiation based on the tartrate-resistant acid phosphatase isoenzyme 5b (TRAP5b) activity, even though they showed approximately the same adhesion of the hMc to the material as PPGC + S 5:5. TRAP5b activity was significantly higher both in the cell lysate (Figure 4a) at PPGC + S 5:5 and also in the medium (Figure 4b). A similar trend was also observed with the resorption markers CatK and CaII (Figure 4c,d), which reached their maximum on day 14/7 and indicated the active resorption activity of the osteoclasts.

### 2.2. Animal Experiments

#### 2.2.1. General Information

A total of 57 of the 60 animals survived the entire observation period. Two animals were lost during anesthesia and 1 died shortly after ovariectomy. Nineteen animals of the empty group, 19 animals of the PPGC + S 3:7 group, and 19 animals of the PPGC + S 5:5 group completed the entire observation period. All wounds healed normally and no serious adverse event was noted.

At the time of euthanasia, we found 4 plate failures in the empty group and three plate failures each in the remaining 19 animals in the PPGC + S 3:7 and PPGC + S 5:5 group, respectively. There were no statistically significant differences found for empty defect vs. PPGC + S 3:7 and PPGC + S 5:5 (*p* = 0.6771). These absolute instable specimens were not used for further analysis. The press-fit implantation of the pre-shaped specimens adapted to the defect was remarkably simple. After implantation of the PPGC + S 5:5 and PPGC + S 3:7 test specimen, no macroscopic rejection or inflammatory reactions and no noticeably increased number of plate fractures indicating bioincompatibility of the implant were observed.

#### 2.2.2. µ-CT Analysis

**Figure 5:**
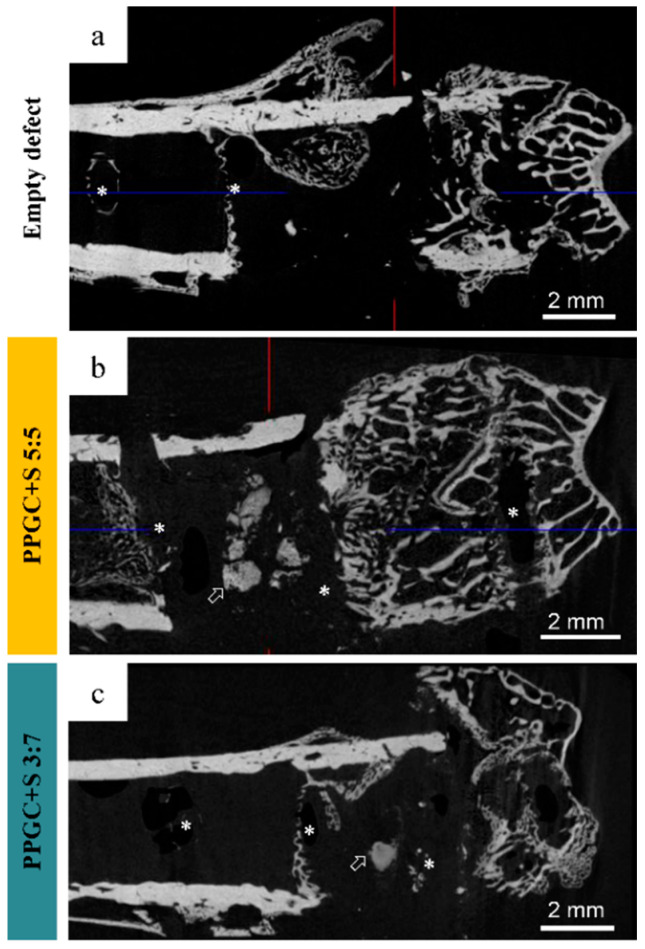
Femora embedded in Technovit scanned on a micro-CT (SkyScan 1173, Bruker microCT). Bone cross-sections without a material (**a**, empty defect), after implantation of PPGC + S 5:5 (**b**) and PPGC + S 3:7 (**c**). The remaining material is indicated by arrows. Asterisks indicate positions of removed screws for T-plate fixation.

Examinations for defect healing were carried out first on the empty defect of osteoporotic animals. The osteoporotic bone status was deduced from the reduced proportion of cancellous bone in the femur based on µ-CT analysis (exemplarily shown in Figure 5) The empty defect in the osteoporotic animal did not heal within six weeks and is, therefore, supercritical. The µ-CT image shows in a two-dimensional representation a large wedge-shaped area without mineral phase, which exhibits an opening of about 4 mm on the bottom side, the side of the osteosynthesis plate. This wedge is not closed at the top but merges into a narrow gap. In the case of PPGC + S 5:5, the amount of remaining material in the defect area was considerably larger than with PPGC + S 3:7. This observation is in line with the change in the material during storage in the cell culture medium (Figure 2). PPGC + S 5:5 showed a curing process by precipitation of calcium phosphate from the surrounding liquid, which causes the material to remain in the defect area, whereas PPGC + S 3:7 showed a continuous decrease in material strength and material dissolution under strontium ion release.

#### 2.2.3. ToF-SIMS

**Figure 6:**
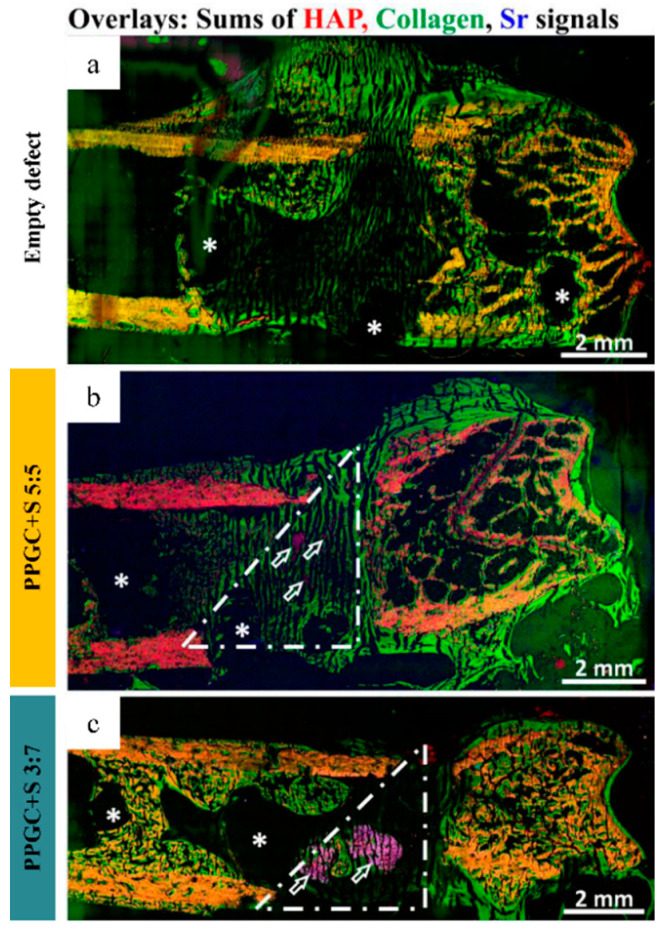
ToF-SIMS of Technovit-embedded sections of explanted femora. Stage scans of the bone cross-sections without a material (**a**, empty defect), after implantation of PPGC + S 5:5 (**b**) and PPGC + S 3:7 (**c**). The RBG overlay mass images show for each sample an overlay of mineralized bone in form of hydroxyapatite (HAP) signals in red, non-mineralized bone areas in form of collagen signals in green, and strontium signals in blue (all signals are listed in Table S1). Thirty kiloelectron volt (keV) Bi^3+^ primary ions were used for ToF-SIMS imaging in positive ion mode. The white lines mark the defect area and the white asterisks show former places of screws. White hollow arrows indicate the remaining material.

Bone sections of 6-8 different animals of each group (empty defect, PPGC + S 5:5, PPGC + S 3:7) were investigated by ToF-SIMS mass spectrometric imaging analysis, performed with a TOF.SIMS 5 instrument (IONTOF GmbH, Münster, Germany), exemplarily shown in Figure S1. In addition, mass spectrometric imaging of one bone section of each group (Figure 5 and Figure 6) was performed with an M6 Hybrid SIMS instrument (IONTOF GmbH, Münster, Germany). ToF-SIMS mass spectrometric imaging analysis with both instruments show for all bone sections areas of mineralized bone in form of characteristic mass fragments of hydroxyapatite (HAP, Figure S1a,g,m), non-mineralized bone in form of collagen signals (Figure S1b,h,n), and strontium signals (Figure S1c,i,o). In RGB overlay images, mass fragments of mineralized bone are shown in red, collagen mass signals are shown in green, and strontium mass signals are shown in blue (Figure 6a–c; Figure S1d,j,p). All used mass signals are listed in Table S1.

RGB overlay images of the empty defect (Figure 5a), PPGC + S 5:5 (Figure 5b), and PPGC + S 3:7 (Figure 5c) reveal the wedge-shaped defect areas as organically filled, non-mineralized areas. Furthermore, ToF-SIMS images of the PPGC + S 5:5 specimen shows that the implanted material was partly degraded after an implantation period of six weeks (Figure 6b; Figure S1g–l). The contours of the inserted specimen are hardly visible in the ToF-SIMS overlay image of the HAP and strontium mass. Nonetheless, still recognizable strontium signals indicate remaining PPGC + S 5:5 material (blue in Figure 6b, marked by white hollow arrows). For the implantation of the PPGC + S 3:7 specimens, the ToF-SIMS image shows strontium signals (blue in Figure 6c) in the former defect area that can be attributed to the remaining specimen (marked with white hollow arrows). These findings are supported by mass spectrometric data with high mass accuracy generated by the Orbitrap mass analyzer, which revealed strontium in areas of still remaining biomaterial of both groups (Figure S2). In both biomaterial groups, strontium signals originate from areas exceeding the defect area, suggesting the incorporation of strontium ions into the surrounding mineralized tissue (Figure S1i,o). This incorporation may have been achieved by osteoblast activity as well as by diffusion and calcium substitution in bone mineral.

**Figure 7:**
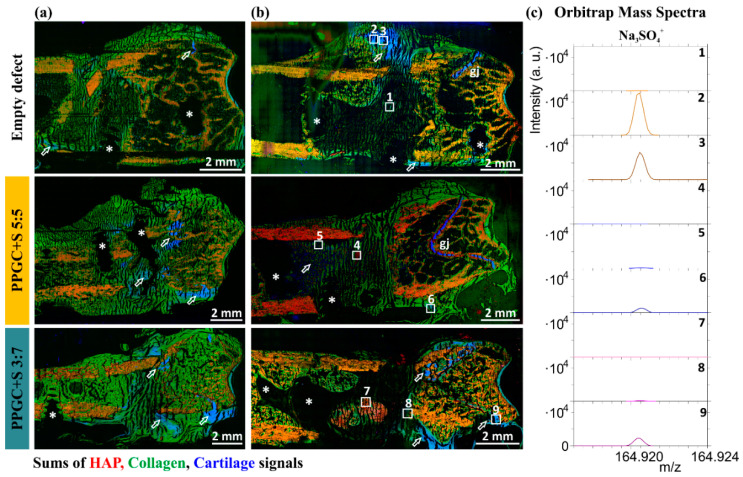
Overview ToF-SIMS mass spectrometric images of the empty group and bone sections containing PPGC + S 5:5 and PPGC + S 3:7 as a biomaterial. RGB overlay images show mass distribution of HAP (mineralized bone, red), collagen mass signals (green) (mass signals listed in Table S1) as well as signals related to cartilage (combination of sulfate signals Na_3_SO_4_^+^ and Na_5_S_2_O_8_^+^) in blue. (**a**) ToF-SIMS mass spectrometric images obtained with TOF.SIMS 5 (IONTOF GmbH, Münster, Germany) and 25 keV Bi^3+^ primary ions. (**b**) 30 keV Bi^3+^ primary ions were used for ToF-SIMS mass spectrometric images obtained with M6 Hybrid SIMS (IONTOF GmbH, Münster, Germany). (**c**) For precise spectrometric measurements, Q Exactive™ orbital trapping mass spectrometer was used with 20 keV Ar_8000_^+^ clusters as primary ion species. Mass spectra were measured on different regions of interest (ROIs) of one bone section of each group (1–9, ROIs are indicated by white rectangles). The white hollow arrows indicate cartilage tissue, white asterisks show former places of screws. Areas of growth joint are indicated by gj.

Additionally, there is organic material in the former defect area of all three groups (empty, defect, PPGC + S 5:5, and PPGC + S 3:7; Figure 7) However, the detection of collagen mass fragments from amino acids glycine, proline, and hydroxyproline, the main components of collagen type 1, alone does not allow conclusions to be drawn about the nature of the tissue or its classification in osteoid or connective tissue. In accordance with our previous study [24], we were able to assign cartilage-related signals (Figure 7 shown in blue in all RGB overlay mass images) detected in newly formed bone tissue to sulfate signals (Figure 7c). Those sulfate signals most likely originating from proteoglycans and are detectable in all bone sections of PPGC + S 5:5 and PPGC + S 3:7 groups within the collagen matrix in areas of the former defect as well as in areas of growth joint (exemplarily shown in two bone sections for each group in Figure 7a,b). In empty defect bone sections, sulfate signals were mainly detectable in areas of outer callus tissue (Figure 7c, orbitrap mass spectra of regions of interest (ROIs) 2 and 3).

#### 2.2.4. Histomorphometry and Histology

Descriptively, an increase in the mineralized tissue (light yellow) was seen in the PPGC + S 3:7 when compared to the other two groups. In addition, there was also an increased chondrocyte activity in PPGC + S 3:7 accompanied by a better bridging of the cortical bone when compared to PPGC + S 5:5 and empty defect group. With respect to biomaterial degradation, no differences were seen in the experimental groups. Further, increased mineralized tissue (as seen in black) depicting better mineralization was seen in the PPGC + S 3:7 group as compared to the other two groups. However, the least mineralized tissue was seen in the case of the empty defect.

**Figure 8:**
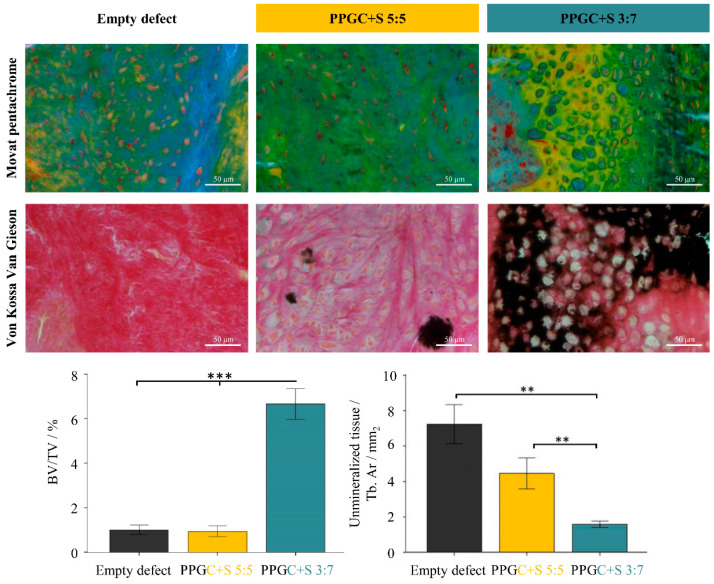
Histological and Histomorphological analysis of Movat pentachrome and von Kossa/van Gieson stained Technovit-embedded sections of the empty defect, PPGC + S 3:7 and PPGC + S 5:5 groups. Pictorial representation of Movat pentachrome staining depicting the highest bone formation and chondrogenic differentiation in PPGC + S 3:7. Moreover, an increase in the mineralized tissue (black) seen in PPGC + S 3:7 as revealed by the von Kossa/van Gieson staining. Histomorphometrical analysis confirming the highest bone formation (BV/TV) and the least unmineralized tissue in PPGC + S 3:7. Asterisks indicate statistical significant differences (* *p* < 0.05; ** *p* < 0.01; *** *p* < 0.001).

Histomorphometric analysis showed a statistically significant increase in bone formation (BV/TV) in the entire defect region in PPGC + S 3:7 when compared to PPGC + S 5:5 and empty group (*p* = 0.0001), respectively (Figure 8). The highest bone formation was seen in the PPGC + S 3:7, which was significantly higher when compared to the PPGC + S 5:5 group (*p* = 0.0001). A simultaneous decrease in the unmineralized tissue (unmineralized tissue/Tb. Ar) was also seen in PPGC + S 3:7 compared to PPGC + S 5:5 (*p* = 0.003) and the empty defect (*p* = 0.001) (Figure 8). Furthermore, a trend was seen where a lower unmineralized tissue was seen in the PPGC + S 5:5 compared to the empty defect alone (*p* = 0.104).

#### 2.2.5. Immunohistochemical Analysis

**Figure 9:**
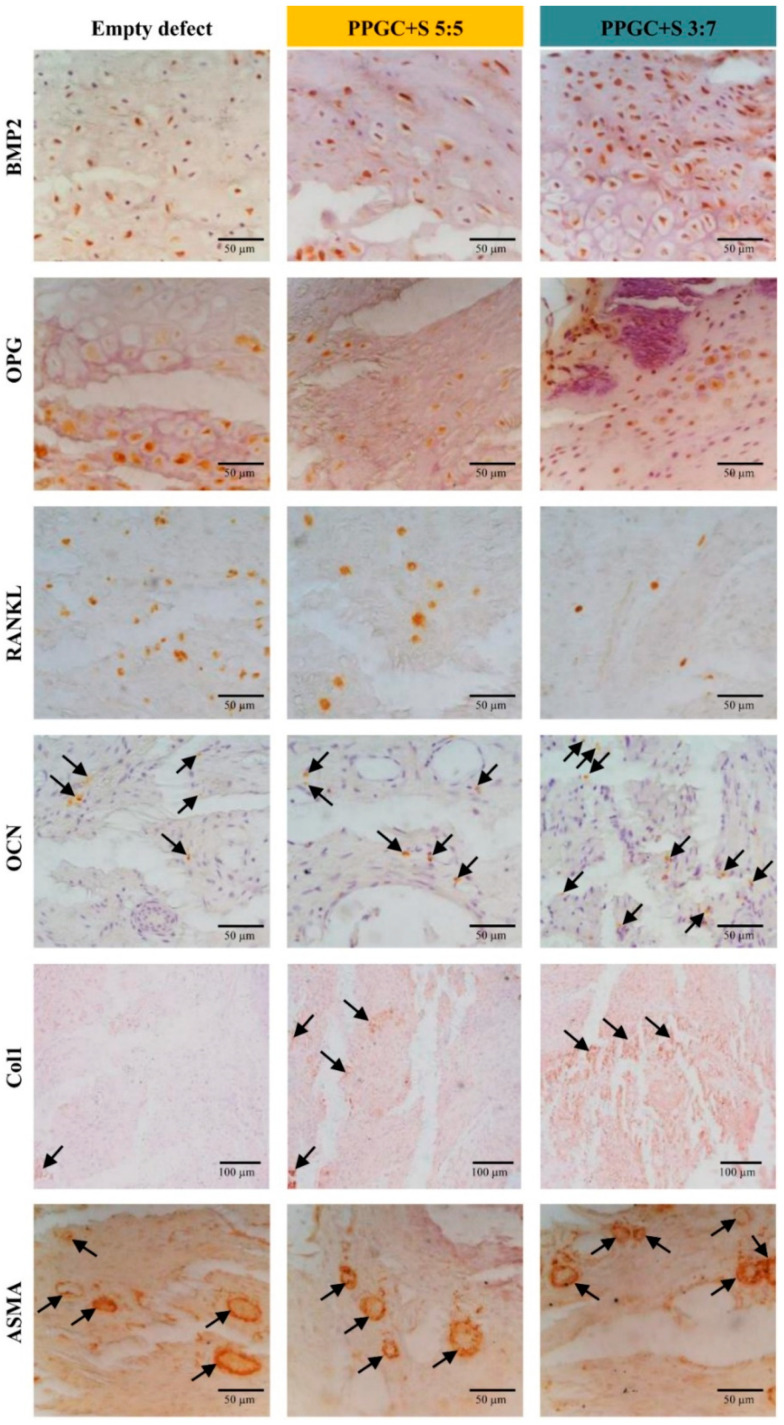
Immunohistochemistry on undecalcified Technovit sections showing expression of potential biomarkers involved in bone formation and resorption. Expression of bone morphogenetic protein-2 (BMP2), osteoprotegerin (OPG); receptor activator of nuclear factor kappa-B ligand (RANKL), collagen type I, and alpha-smooth muscle actin (ASMA) are shown in the ROI for the empty defect as well as PPGC + S 5:5 and PPGC + S 3:7. Additionally, OPG expression is shown in an overview. Only dark brown stains for osteocalcin (OCN) and type I collagen (Col1) marked with black arrows were used for analysis (to avoid the analysis of non-specific staining). For ASMA, blood vessels larger than the size of a cursor head were taken into count to avoid bias (indicated by arrows). A histomorphometrical analysis of the biomarker expression is provided in Figure 10.

**Figure 10:**
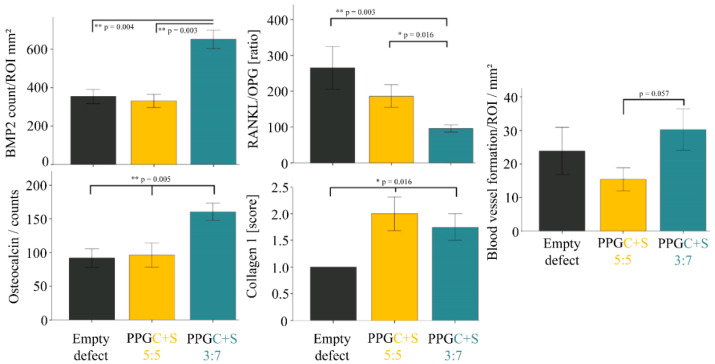
Histomorphometrical analysis of the biomarker expression of Figure 9 is provided for a better comparison of the three groups. Asterisks indicate statistical significant differences.

Immunohistochemical staining revealed an increased positive bone morphogenic protein-2 expression in the PPGC + S 3:7 group compared to PPGC + S 5:5 and empty defect group, respectively. In addition, the expression of RANKL/osteoprotegerin (OPG) ratio, an important determinant of bone resorption, was found to be the lowest in the PPGC + S 3:7 group compared to PPGC + S 5:5 (*p* = 0.003) and empty defect group (*p* = 0.004) (Figure 9 and Figure 10). The analysis of new blood vessel formation using alpha-smooth muscle actin antibody revealed a comparatively higher number of positively stained vessels in the PPGC + S 3:7 compared to PPGC + S 5:5 alone (*p* = 0.057). Evaluation of the expression of osteocalcin, an important biomarker of bone formation, revealed the highest expression in PPGC + S 3:7 compared to the other two groups (*p* = 0.005). To further support this data, the evaluation of collagen type 1, which forms the major organic component of the ECM in bone, showed an increased expression in PPGC + S 3:7 and PPGC + S 5:5 compared to the empty defect (*p* = 0.016). However, no significant differences in the collagen type 1 expression were seen between PPGC + S 3:7 and PPGC + S 5:5. The highest ED1 activity was seen in the PPGC + S 5:5 group compared to PPGC + S 3:7 (*p* = 0.003) and empty defect (*p* = 0.004), respectively (Figure S3 provided in supplementary).

#### 2.2.6. Molecular Biology

**Figure 11:**
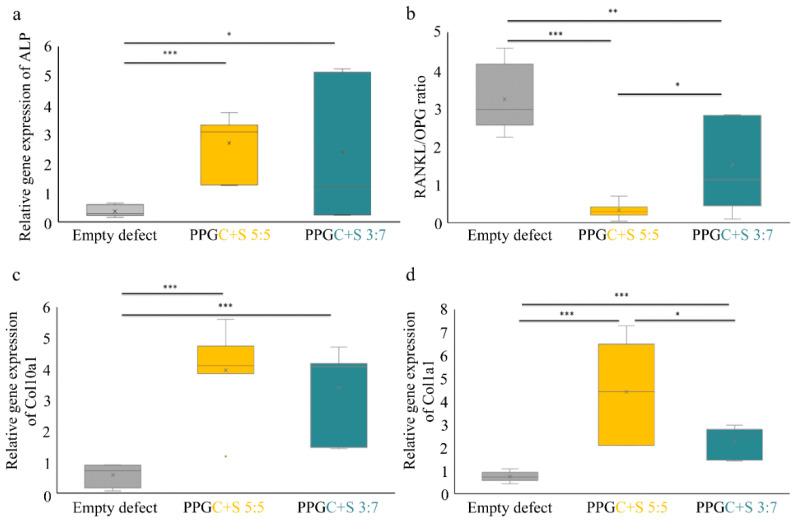
Gene expression analysis of ALP, RANKL/OPG, Col10a1, and Col1a1. Asterisks indicate statistical significant differences (* *p* < 0.05; ** *p* < 0.01; *** *p* < 0.001).

An increased expression of ALP, an osteoblast marker, was seen in the PPGC + S 3:7 (*p* = 0.0273) and PPGC + S 5:5 (*p* = 0.0001), indicating better mineralization of bone when compared to empty defect. RANKL/OPG ratio, an important determinant of osteoclastogenesis, was seen to be the lowest in PPGC + S 5:5 compared to empty defect (*p* = 0.0001) and PPGC + S 3:7 (*p* = 0.0.0128) (Figure 11). Furthermore, a significantly lower RANKL/OPG ratio was seen in the PPGC + S 3:7 compared to empty defect alone (*p* = 0.0076). Expression of Col10a1 was significantly higher in the PPGC + S 3:7 (*p* = 0.00023) and PPGC + S 5:5 (*p* = 0.0001) compared to the empty defect group. However, no differences in Col10a1 expression was seen in between the test groups. In addition, Col 1a1 in PPGC + S 5:5 was significantly higher than that of the PPGC + S 3:7 (*p* = 0.014) and empty defect group (*p* = 0.0001). Further, a significantly higher expression of Col1a1 was seen in PPGC + S 3:7 compared to the empty defect group (*p* = 0.0001).

## 3. Discussion

For the first time, the materials used in this study have been subjected to in vitro co-cultivation of osteoblasts and osteoclasts as well as in vivo animal testing. This allowed the direct analysis of the influence of the materials on the osteoblast/osteoclast ratio in both cases. First of all, the influence of the material composition on the ion concentration during material storage in the cell culture medium was investigated. The higher strontium content in PPGC + S 3:7 caused an initial ion release but led over the whole time of incubation to the maintenance of the total cation concentration on the initial level of the medium (1.8 mM). At the same time, material degradation took place, which was reflected by the decrease of the indirect tensile strength of the specimens. This was in line with the observations previously made for this class of materials, ranging from strontium-free PPGC + S 10:0 to calcium-free PPGC + S 0:10 [12,13], upon which the selection of materials for co-culture and animal testing was based. With PPGC + S 5:5, the following effect occurred during incubation in the cell culture medium. After an initial release of ions, a significant reduction of the cation concentration was present. This was attributed to mineral phases precipitating from the medium on PPGC + S 5:5, which is also referred to as bioactivity. As a result, an increase in strength of the specimens was measured, which follows the rules of calcium phosphate cement hardening [25,26]. Therefore, it can be concluded for further discussion that in the case of PPGC + S 3:7, an increased ion release and degradation occurs compared to the highly bioactive PPGC + S 5:5.

Based on an almost equivalent adhesion of the hBMSC, a significantly increased differentiation to osteoblasts was shown when cultivating hBMSC on higher strontium containing PPGC + S 3:7. Evidence of the pro-osteogenic effect of elevated strontium concentrations on osteoblasts in vitro has been published earlier [9,12,27,28]. At the end of co-culture, a well-spread actin network of osteoblasts was observed on both materials. This hMSC/osteoblasts layer was also seen from SEM images of the material’s surface. Occasionally, multinucleated, rather round-shaped, osteoclast-like cells were visible between the stretched osteoblasts on the material surface with cLSM and SEM. Neither osteoblast nor osteoclast differentiation additives was added to the co-culture after the addition of hMc to the pre-differentiated osteoblasts. Therefore, the osteoclast differentiation was the focus of attention to identifying the material inherent properties such as physicochemical dissolution and ion release as a key influence on the osteoblast/osteoclast ratio. The significant inhibitory effect on osteoclasts in vitro of the material with increased strontium content and faster degradation (PPGC + S 3:7) is in accordance with the literature. Various mechanisms have been described, as there is the dose-dependent inhibitory effect of strontium ions on osteoclast differentiation by downregulation of RANKL-induced osteoclastogenesis and activation of the calcium-sensing receptor (CaSR), which was shown by Caudrillier et al. using RAW 264.7 cells and PBMCs [29]. Furthermore, the CaSR was shown to be a target of strontium ions in mature rabbit osteoclasts to induce apoptosis of these cells [30]. Moreover, a decrease in TRAP activity and disruption of cytoskeleton organization after strontium ion treatment of murine osteoclasts was described by Bonneleye et al. [28]. Therefore, it is well accepted that strontium ions decrease bone resorption by decreasing osteoclast activity and stimulates new bone formation by promoting osteogenic differentiation [9,31].

The results of the in vivo investigations confirm the conclusions of the in vitro degradation and cell culture experiments. Accordingly, defect healing can be promoted with the help of strontium ions even in the case of osteoporosis. Impaired bone remodeling was taken into account in material development by focusing on bone cell stimulation. The degradable bone substitute materials presented here are suitable for this purpose by locally releasing their components, in particular strontium ions. Since PPGC + S 5:5 cures to a calcium phosphate phase, a comparatively small amount of remaining material is observed in the ToF-SIMS images based on the strontium signal. This is due to the fact that strontium is released into the environment during the dissolution and reprecipitation process of the initially strontium-substituted brushite, while a calcium phosphate with increased strength is formed, which can be identified particularly well in the µ-CT images. Even though PPGC + S 5:5 has a less rapid decrease in mass in vivo owing to its bioactivity, the Sr^2+^ ions are released during the in vivo experiment and incorporated into existing or newly formed bone tissue, as shown by ToF-SIMS. These findings of strontium incorporation in bone tissue after release from bone cements are in line with previous ToF-SIMS studies of strontium containing bone cements [32,33]. The lower initial amount of strontium in PPGC + S 5:5 caused a lower anti-osteoclastic effect than PPGC + S 3:7 but still a good stimulation of new bone formation. PPGC + S 3:7 was shown to have a faster degradation, with a high strontium-associated anti-osteoclastic effect and an increased osteoblastic reaction towards the new bone formation. It is to be assumed that the degraded material serves as a stimulus for bone regeneration after degradation and that the bone-like components can also be used for the remodeling. Of particular value is that this finding applies to the osteoporotic defect, which in turn should also support the healing of defects in the case of healthy bone. In addition to monitor release and incorporation of Sr^2+^ ions, we were able to detect mass fragments for cartilage tissue with both ToF-SIMS and Orbitrap-SIMS analysis. In accordance with a previous study [24], we assume these signals, which were detected in newly formed bone tissue as well as areas of growth joint, to originate from proteoglycans.

In the in vivo experiments, the circulating OPG level was increased and the circulating RANKL level was decreased in the case of PPGC + S 3:7 compared to those of PPGC + S 5:5 as revealed by the immunohistochemical analysis. The most significant difference was found for the RANKL/OPG ratio. The RANKL-to-OPG balance is of critical importance for bone remodeling and the preservation of bone mass. The osteoclast activity is mostly dependent on the relative balance of RANKL and OPG, which is why it has been suggested that the serum RANKL-to-OPG ratio is of rather more importance than individual protein concentrations and is the critical factor for determining osteoclastic activation at the bone level. Any alterations in this ratio have been associated with several bone loss diseases. In accordance with this, the lowest RANKL/OPG ratio was seen in the PPGC + S 3:7, which could be attributed to the addition of strontium. In addition, pro-osteogenic proteins osteocalcin and BMP-2 are significantly increased, which furthermore supports an enhanced osteoblast/osteoclast ratio as shown in in vitro co-culture.

With the help of Movat pentachrome staining, a large number of chondrocytes in the defect area were detected in histological sections. The effect of strontium on chondroblasts has not yet been investigated in detail. However, the analyses by Henrotin et al. and Yu et al. [34,35] suggest that strontium can serve as a starting point for osteo-chondral bone formation. Stimulation of this bone formation process, similar to early childhood tissue development, could open a new path for defect/fracture healing for the regeneration of large bone defects.

## 4. Materials and Methods

### 4.1. Mineral Precipitation and Specimen Preparation

Preparation and material scientific characterization of the composite materials have been published earlier and are presented here briefly [11,12,13]. The gelatin-modified calcium/strontium phosphates were synthesized by precipitation as follows. First, porcine gelatin (300 bloom, 20 mesh, Gelita, Eberbach, Germany) was suspended in deionized water (0.9%) and liquefied by heating in a water bath (50 °C). Afterward, a 0.106 M Na_2_HPO_4_ (Roth, Karlsruhe, Germany) solution was added to the gelatin and the pH was adjusted with 3 M HCl to 7.0 ± 0.1. The phosphate pre-structuring of gelatin occurred during 8 h storage, before a 1 M cationic mineralization solution was added. The mineralization solutions were prepared of CaCl_2_·2H_2_O and SrCl_2_·6H_2_O (Roth) in the molar ratio of calcium to strontium of 5:5 and 3:7. The solutions were added with 2.5 mL/min. After the addition of the solutions, the mineral suspension was stirred for 3 h. The resulting gelatin-modified minerals are named PPGC + S 5:5 and PPGC + S 3:7, respectively, as an abbreviation for phosphate pre-structured gelatin mineralized with calcium (C) and strontium (S) with the above-mentioned ratios of calcium to strontium. After ripening, the mineral was separated from the supernatant by centrifugation (3000 rcf, 5 min). It is important to mention that the final minerals contain a measured calcium/strontium mass ratio of 63%/37% for PPGC + S 5:5, which is a brushite with disturbed morphology and crystal structure by strontium substitution, while PPGC + S 3:7 is a strontium phosphate slightly doped with calcium ions showing a measured calcium/strontium ratio of 10%/90% [12,13].

After mineral precipitation, the preparation of porous specimens for cell culture and in vivo tests was performed. Therefore, 4 g of the mineral pellet obtained after ripening and centrifugation was resuspended in 2 mL 0.5 M HEPES buffer (pH = 8.0, Roth) and 1 mL cross-linking solution prepared from 300 mM 1-Ethyl-3-(3-dimethylaminopropyl)carbodiimide (EDC, Sigma-Aldrich, Darmstadt, Germany) and 150 mM N-Hydroxysuccinimide (NHS, Sigma-Aldrich). The suspension of mineral, HEPES, and cross-linker was mixed thoroughly and transferred to specially prepared casting molds, which were placed in 48-well plates and centrifuged afterward (1900 rcf, 10 min) for sedimentation and cross-linking. The casting molds for in vitro test specimens were custom-made hollow cylindrical PTFE inserts with an inner diameter of 6.5 mm, in which 450 µL of the suspension was transferred. For the in vivo tests, a silicone mold was custom-made to produce a cylindrical test specimen with an ellipsoidal base (major axis = 4 mm, minor axis = 2.8 mm) according to the established defect geometry of Alt et al. [16] and according to the approximate cross-sectional geometry of a rat femur.

After lyophilization, the specimen was cut manually with a scalpel to a height of 3.5 mm for cell culture and 4 mm for implantation in the femur defect. Finally, all specimens were sterilized by gamma irradiation at 25 kGy before used for implantation.

### 4.2. In Vitro Analysis of Ion Release and Cellular Reaction in Co-Culture

#### 4.2.1. Ion Release and Mechanical Strength

To investigate ion release and mechanical strength during incubation, 6 specimens (d = 6.5 mm, h = 3.5 mm) were stored in 8 mL of cell culture medium for 28 d at 37 °C. A-minimal essential medium (α-MEM, Biochrom, Darmstadt, Germany) was supplemented with 10% fetal calf serum (FCS, Biochrom), 100 U/mL penicillin, 100 µg/mL streptomycin (1% penicillin/streptomycin, Biochrom), and 2 mM L-glutamine. Calcium and strontium ion concentrations were measured after 1, 14, and 28 days as a combined cation concentration using a colorimetric Fluitest^®^ CA CPC test kit (Analyticon, München, Germany) and an Infinite 200 Pro microplate reader (Tecan, Männedorf, Switzerland). Indirect tensile strength was measured for 6 specimens at the respective time points. The tests were performed with an Instron 5566 (Instron, Norwood, UK) universal testing machine at 0.5 mm/min, and the maximum force leading to fracture was measured to calculate indirect tensile strength.

#### 4.2.2. Osteoblast/Osteoclast Co-Culture

The co-cultivation of osteoblasts and osteoclasts by the pre-cultivation and differentiation of human bone marrow stromal cells (hBMSC) to osteoblasts and the addition of human monocytes (hMc) after 7 d without the addition of further osteoblastic and osteoclastic differentiation factors to perform biomaterial characterization has been extensively described and analyzed earlier [22,36,37]. In short, the co-culture is performed as follows. hBMSCs, isolated from bone marrow aspirates, were kindly provided by Professor Bornhäuser and co-workers, Medical Clinic I, Dresden University Hospital [38]. The cells were expanded in Dulbecco’s modified Eagle’s medium (DMEM), low glucose, supplemented with 10% fetal calf serum (FCS), 100 U/mL penicillin, 100 μg/mL streptomycin in a humidified atmosphere at 37 °C and 7% CO_2_. Medium and all supplements were obtained from Biochrom. hMc were isolated from human buffy coats using a modified OptiPrep™ (ProGen Biotechnik, Heidelberg, Germany) density-gradient medium technique described in detail previously [22,37]. The monocyte-enriched PBMC fraction was collected and hMc were purified via magnetic-activated cell sorting by negative selection using a Monocyte Isolation Kit II (Miltenyi, Bergisch Gladbach, Germany). For the co-culture experiments, the medium was changed to alpha-MEM supplemented with 7.5% heat-inactivated FCS, 7.5% human serum (AB, off the clot), 100 U/mL penicillin, 100 μg/mL streptomycin, and 50 μM ascorbic acid 2-phosphate. Sterilized specimens were pre-incubated in co-culture-medium for 24 h before cell seeding. hBMSC in passage 5 were seeded at a density of 2·10^4^ per specimen in 48-well plates using custom-made PTFE inserts to reduce the well size during seeding to that of 96-well plates. After adhesion for 24 h, the inserts were removed and the specimen transferred to fresh 48-well plates in order to exclude cells adherent to the well plates. Osteogenic differentiation was started on day 3 by the addition of 10 nM dexamethasone (Sigma-Aldrich) and 10 mM β-glycerophosphate (Sigma-Aldrich) to the medium.

hMc were added via drop seeding on day 7 with 2·10^5^ hMc per specimen using the custom-made PTFE inserts again. Osteogenic additives such as dexamethasone and β-glycerophosphate were excluded with the addition of hMc. After adhesion for 24 h, the specimens were transferred to fresh 48-well plates in order to exclude non-adherent hMc. Co-cultivation was performed for additional 3 weeks, with medium changed twice a week. Reference monoculture of hBMSC was cultivated identically to the method described above without the addition of hMc. Finally, the medium was removed and the scaffolds were washed and frozen at –80 °C until biochemical analysis. Scaffolds for microscopic analysis were fixed with 3.7% formaldehyde (FA) in PBS and stored at 4 °C in 0.37% FA/PBS.

#### 4.2.3. Biochemical Analyses of Co-Culture, Confocal Laser Scanning Microscopy (cLSM) and Scanning Electron Microscopy (SEM)

For all biochemical analyses, cell lysates were prepared with 1% Triton X-100 (Sigma-Aldrich) in PBS. A SpectraFluor Plus microplate reader (Tecan) was used for colorimetric measurements. Adhesion von hBMSC was determined via the total activity of lactate dehydrogenase (LDH) activity using the Cytotoxicity Detection Kit (Takara). The absorbance was read at 492 nm and LDH activity was correlated with the cell number by analyzing cell lysates of defined cell numbers.

Osteoblast differentiation was evaluated by alkaline phosphatase (ALP) activity. Cell lysates were added to ALP substrate buffer, containing 2 mg/mL p-nitrophenyl phosphate (Sigma), 0.1 M diethanolamine, 1 mM MgCl_2_ (Sigma-Aldrich), and 0.1% Triton X-100 (pH 9.8), and the mixture was incubated at 37 °C for 30 min. The enzymatic reaction was stopped by the addition of 0.5 M NaOH, and absorbance was measured at 405 nm and compared to different concentrations of p-nitrophenol.

Adhesion of hMc on (already with osteoblasts pre-cultivated) materials was determined by measurement of DNA. Examination of DNA amount was carried out using the Quant-iT™ PicoGreen® dsDNA Reagent, measuring the fluorescence intensity (excitation/emission) at 485/535 nm and correlated with a calibration line of calf thymus DNA (Sigma-Aldrich).

Osteoclast differentiation was evaluated by enzyme activity measurements of tartrate-resistant acid phosphatase (TRAP5b), carbonic anhydrase II (CAII), and cathepsin K (CTSK). TRAP5b analysis was performed intracellular (cell lysates) and extracellular (cell culture supernatants) using naphthol–ASBI phosphate (N-ASBI-P, Sigma-Aldrich) as a substrate according to Janckila et al. [39]. Cell lysates or supernatants were added to TRAP5b substrate buffer, containing 2.5 mM N-ASBI-P in 100 mM Na-acetate (Sigma) buffer with 50 mM Na-tartrate (Sigma-Aldrich), 2% NP-40 (Sigma-Aldrich), and 1% ethylene glycol monomethyl ether (Sigma-Aldrich) at pH 6.1, and the mixture was incubated at 37 °C for 1 h. The reaction was stopped using 0.1 M NaOH, and fluorescence intensity (excitation/emission) was measured at 405/535 nm and correlated to a TRAP5b standard. For CaII activity measurement, cell lysates were incubated with 2 mM 4-nitrophenylacetate (Sigma-Aldrich), 12.5 mM TRIS (pH 7.5), and 75 mM NaCl, and absorption at 400 nm was measured after 5 min reaction time. A calibration line with different concentrations of 4-nitrophenol was used as a correlation. For CTSK activity measurements, cell lysates were mixed with a substrate solution of 100 μM Benzyloxycarbonyl-L-leucyl-L-arginine 4-methylcoumaryl-7-amide (peptide) in 100 mM sodium acetate (Roth), 4 mM EDTA, and 4 mM dithiotreitol (molecular probes), pH 5.5. After 30 min at 37 °C, the reaction was stopped with 100 mM iodoacetic acid sodium salt (Fluka) in 0.1 M Tris (pH 8). Fluorescence intensity (excitation/emission) was measured at 365/440 nm and correlated with different concentrations of 7-amino-4-methylcoumarin (AMC, Sigma-Aldrich).

CLSM was used to visualize hBMSC and hMc, and to evaluate their adhesion as well as proliferation (hBMSC) and differentiation (hMc). Fixed cells were permeabilized with 0.2% Triton-X-100 in PBS and blocked with 1% BSA for 30 min. Cytoskeletal actin was stained with AlexaFluor 488-Phalloidin (life technologies) and cell nuclei were stained with 4′,6-diamidino-2-phenylindole (DAPI; Sigma-Aldrich). Microscopy was performed on an upright Axioscop 2 FS Mot with an LSM 510 META module (Carl Zeiss, Jena, Germany) equipped with an Ar + laser for Alexa Fluor 488 excitation and a NIR-fs-laser for excitation of DAPI at 750 nm (2-photon excitation).

SEM preparation of cell-seeded and fixed specimens was performed by dehydration with a graded ethanol series and subsequent infiltration with hexamethyldisilazane (Fluka). The samples were coated with carbon using an SCD 050 coater (Balzers, Liechtenstein), and microscopy was performed on an ESEM XL30 (FEI, Hillsboro, OR, USA) in Hi-Vac mode with 3 kV acceleration voltage.

### 4.3. Ethics Statement and Animal Study

After approval of the animal experiments by the local authorities according to the Protection of Animals Act (Ref. number: V54–19c 20/15–FU/1121), 60 female Sprague-Dawley rats (10 weeks old) were used, 10 for µ-CT histological and ToF-SIMS analysis and 10 for molecular analysis (per group). The animals were kept in filter-topped plastic cages (4 rats per cage) that allowed free access to food and water. Rooms were maintained at 22 °C and 40–60% humidity. The rats were randomly assigned to three different treatment groups: (1) sham (*n* = 20), (2) PPGC + S 5:5 (*n* = 20), and (3) PPGC + S 3:7 (*n* = 20).

**Figure 12:**
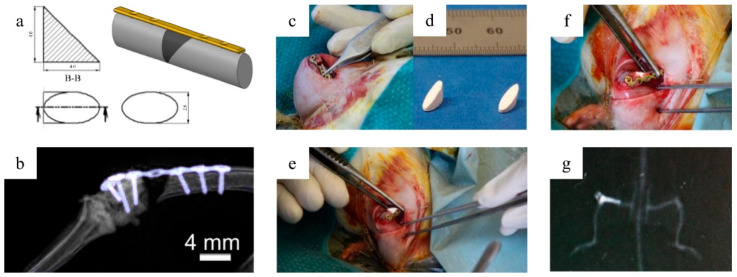
Schematic representation of the wedge-shaped metaphyseal femoral defect in the osteoporotic rat model (**a**); micro-computer tomography of the empty defect according to Alt et al. [16] (**b**); photographs of the blank defect (**c**) and the cut specimen (**d**) as well as defect filling (**e**,**f**) and post-operative bone density measurement by dual X-ray absorptiometry (**g**).

The osteopenic bone status, characterized by a decreased mineral density, is a preliminary bone status of osteoporosis in the selected animal model and was induced by bilateral ovariectomy combined with a multi-deficient diet as described previously [16]. In brief, under general anesthesia with 4 vol.% isoflurane for induction and 1 vol.% during surgery and buprenorphine (0.01 mg/kg bodyweight), bilateral ovariectomy was performed. The animals were allowed two weeks of recovery and were then fed with calcium-, phosphorus-, and vitamin D3-, soy- and phytoestrogen-free diet (10 mm pellets, Altromin-C1034, Altromin Spezial futter GmbH, Lage, Germany) for 12 weeks. A wedge-shaped fracture-defect was created (Figure 12) with a length of 4 mm and a medial gap of 0.35 mm at the distal metaphysis of the left femur using an ultrasound bone saw (Piezosurgery^®^ 3, Saw blade OT7S-3, Mectron, Köln, Germany). The defect was stabilized by a T-shaped 7-hole mini-plate (Leibinger^®^XS-miniplate, Stryker, Kalamazoo, MI, USA) as described by Alt et al. [16,40]. The defects were filled either with a pre-shaped specimen of PPGC + S 5:5 or PPGC + S 3:7 (Figure 12d–f) or left empty (Figure 12c). The multi-deficient diet was continued until euthanasia, i.e., 6 weeks after femur surgery. Measurement of bone mineral density (BMD g/cm2) by means of dual-energy X-ray absorptiometry (DXA, Figure 12g) using DXA scanner (Lunar prodigy, GE Healthcare, Chicago, IL, USA) was performed to ensure the bone status. In the case of plate fixation failure, e.g., breakage or loosening, specimens were not taken to further analysis.

### 4.4. µ-CT Analysis and ToF-SIMS

Femora embedded in Technovit were scanned on a micro-CT System manufactured by Bruker micro-CT (SkyScan 1173, Bruker microCT, Kontich, Belgium). Details concerning the scanning procedure are displayed in Table 1 according to the guidelines for the assessment of bone microstructure described by Bouxsein et al. [41]. A 1 mm Aluminium filter was used for beam filtration, leading to reduced beam hardening artifacts. Scanning time was 1 h 36 min. Reconstructions were carried out using the NRecon-Software Version 1.7.0.4 (Bruker microCT, Kontich, Belgium), resulting in images of 8bit grayscale. Beam hardening correction was escalated to 45%. A Gaussian filter (smoothing kernel = 2, smoothing = 1) was employed for image reconstruction.

For mass spectrometric imaging, the embedded bone sections (5 µm thick) were deplastified with 2-methoxyethylacetate (MERCK, Darmstadt, Germany) three times for 20 min each, prior to analysis. Bone sections of 6–8 different animals of each group (PPGC + S 5:5, PPGC + S 3:7, empty defect; exemplarily shown in Figure S1, Figure 7) were investigated by ToF-SIMS imaging analysis, performed with a TOF.SIMS 5 instrument (IONTOF GmbH, Münster, Germany), equipped with a bismuth primary ion source (25 keV). All measurements were carried out in the positive ion mode and charge compensation was done with a low energy electron flood gun. Image data were obtained by so-called stage scans in the high-current bunched mode with Bi^3+^ ions as primary ion species. Scanning was performed in sawtooth mode with the following parameters: 10 shots per pixel, 10 frames per patch of 400 × 400 µm² and 100 pixels/mm, cycle time 55 µs and 3 scans, with primary ion currents of 0.5–0.7 pA. For mass calibration, the following signals were used: H^+^, H_2_^+^, CH_3_^+^, Na^+^, CH_4_N^+^, Ca^+^, C_4_H_8_N^+^, and C_5_H_12_N^+^. Obtained mass resolution *m/*Δ*m* (FWHM) was for all measurements better than 3000 for the Ca^+^ mass signal.

In addition, mass spectrometric imaging of one bone section of each group (Figure 6 and Figure 7) was performed with an M6 Hybrid SIMS instrument (IONTOF GmbH, Münster, Germany), equipped with a bismuth primary ion source (30 keV). M6 Hybrid SIMS is equipped with a combination of a TOF analyzer and a Q Exactive™ orbital trapping mass spectrometer. For overview mass imaging of bone sections, the ToF analyzer was used. Scanning was performed in sawtooth mode with the following parameters listed in Table 2.

During the entire measurements, oxygen gas flooding and low energy electron flood gun were used for charge compensation. The following signals were used for internal mass calibration: CH_3_^+^, Na^+^, CH_4_N^+^, Ca^+^, C_4_H_8_N^+^, and C_5_H_12_N^+^. Obtained mass resolution *m/*Δ*m* (FWHM) was better than 3000 for the Ca^+^ mass signal.

For spectrometric measurements, Q Exactive™ orbital trapping mass spectrometer was used. Mass spectra were measured on different ROIs of one bone section of each group (Figure 7). Parameters for Orbitrap spectra measurements are listed in Table 3.

Orbitrap mass calibration was performed once at the beginning of each measurement session using Ag clusters between Ag_1_ and Ag_15_ from a reference sample. As the mass calibration remains stable for >25 h, no recalibration using known peaks from the spectrum was necessary.

Data evaluation of all measurements was done with Surface Lab 7.1 software (IONTOF GmbH, Münster, Germany). A detailed prove of ToF-SIMS for bone imaging was described previously [24,42].

### 4.5. Sample Processing, Staining Procedures, and Histomorphometry

Harvested femurs were fixed in phosphate-buffered 4% paraformaldehyde for 48 h at 4 °C until processing. Samples were embedded in Technovit^®^ 9100 NEU according to the manufacturer’s protocol (Heraeus Kulzer, Hanau, Germany) and sectioned into 5 µm thick slices. Qualitative and quantitative morphological analyses were performed on sections stained with movat-pentachrome and von Kossa/van Gieson as described earlier [43,44]. For histomorphometric analysis of the osteoid formation, new bone formation, implant retention, and macrophage count, the original ROI was used comprising the initial wedge-shaped osteotomized defect area. Adobe Photoshop CS6 was used for measurements for ROIs, area of bone, osteoid, implant, and the void (sectioning artifacts) to determine the ratio of bone volume versus tissue volume (BV/TV), implant retention in the defined ROI, and osteoid volume versus tissue volume (OV/TV). For histomorphometric evaluations, there were two regions of interest (ROIs). The first ROI was made by directly tracing over the material followed by 100 pixels increase to include the biomaterial–tissue interface. The second ROI consisted of the entire initial wedge-shaped osteotomized defect area to assess the new bone formation. With the help of Adobe Photoshop CS6, the measurements for the area of bone (yellow-stained section), ROIs, implant (black), and the void (colorless gaps), unmineralized tissue/osteoid (dark pink), were made, respectively, to determine bone versus tissue ratio (BV/TV).

Count for ED1-positive cells per trabecular area (Macrophage/Tb.Ar) was also performed. The measurements were done blindfolded concerning the test groups. The consecutive sections were then used for immunohistochemical and ToF-SIMS analysis.

### 4.6. Immunohistochemistry

Immunohistochemistry was carried out with the following antibodies: Rabbit Anti-BMP2 Polyclonal Antibody (AP20597PU-N; Acris), Rabbit Anti-Osteoprotegerin Polyclonal Antibody (250800; Abbiotec), Rabbit Anti CD254/RANKL Polyclonal Antibody (AP30826PU-N; Acris), Monoclonal Mouse Anti-Human Muscle Actin (M0635; Dako), and Mouse Anti-Rat Monocytes/Macrophages Monoclonal Antibody ED1 (MAB1435; Chemicon), respectively.

Goat Anti-Rabbit (BA-1000, Vector) was used as a secondary antibody for BMP-2, OPG, and RANKL followed by a Vectastain ABC kit (Elite PK-6100, Standard, Vector Laboratories, Burlingame, CA, USA). Finally, visualization was done using Nova Red (SK4800, Vector Laboratories, Burlingame, CA, USA), and hematoxylin (Shandon Inc., Pittburgh, PA, USA) was used as a counterstain. For ED1 and ASMA, antigen identification was done using DakoEnvision+System-HRP (DAB) for use with Mouse Primary Antibodies (Dako, K4006). Images were taken using Axioplan 2 Imaging system (Carl Zeiss, Jena, Germany) with a Leica DC500 camera (Leica, Bensheim, Germany), acquired with Leica IM1000 software and processed using Adobe Photoshop CS6.

### 4.7. mRNA Preparation and Gene Expression Analysis

The gene expression analysis was carried out for the following target genes as described earlier:

(A) For new bone formation: 1. Alkaline phosphatase (ALP), an osteoblast marker indicating bone mineralization; 2. Osteocalcin (OCN), a noncollagenous protein secreted by osteoblasts, which plays a role in mineralization and calcium ion homeostasis; 3. Collagen type10 alpha1 (Col10α1), a hypertrophic chondrocytes marker; 4. Runt-related transcription factor 2 (Runx2), an essential protein for osteoblastic differentiation; 5. Collagen type I alpha1, a major component of type I collagen, (Col1α1).
(B) For bone resorption: 1. TNFSF11gene (RANKL, RANK ligand) as a member of the tumor necrosis factor (TNF) cytokine family, ligand for osteoprotegerin, and a key factor, which regulates osteoclast differentiation and activation; 2. TNFRSF11B gene (osteoprotegerin; OPG), a decoy receptor for RANKL that works by neutralizing its function in osteoclastogenesis; 3. Carbonic anhydrase, an osteoclast marker involved in bone matrix dissolution. β2-microglobulin (B2M) was used as a reference gene. The primer pairs are provided in the Supplemental Table S2.

### 4.8. Statistical Analysis

The data were checked for normal distribution and homo-scedasticity. One-way ANOVA along with Tukey’s multiple comparison tests was applied to determine variation between the groups in histomorphometrical analyses. If the requirements were not complied, nonparametric Mann–Whitney U test was performed. All the statistical analysis was done using SPSS V. 20.0 (SPSS Inc., Chicago, IL, USA) or OriginPro 2017 (OriginLab Cooperation). The asterisks and hashes indicate the significance level *^,#^ *p* < 0.05, **^,##^ *p* < 0.01, and ***^,###^ *p* < 0.001, respectively.

## 5. Conclusions

The crucial question, whether a bone substitute material can be adapted to the requirements of an osteoporotic-like condition was answered by in vitro cell culture and subsequent in vivo experiments. The present study confirms the potential of strontium-substituted calcium phosphates for influencing the development and activity of osteoblasts and osteoclasts in the same manner in vitro as well as in vivo. The cellular reactions in vitro, such as the support of osteoblast differentiation by calcium and strontium phosphates, as well as the impairment of osteoclast maturation owing to comparatively high strontium contents, are directly and indirectly linked to the structure of the biomaterials and their degradation. The in vivo defect healing was promoted with the help of the high strontium-containing material. This is of particular value as this finding applies to the osteoporotic defect, wherefore in turn the material should also support the healing of defects in the case of healthy bone. The degradable bone substitute materials are suitable for this purpose by locally releasing their components, in particular the strontium. In the future, gelatin-modified calcium/strontium phosphates might be a promising treatment strategy for patients in need of surgical care of large bone defects.

## Figures and Tables

**Table 1:** Scanning parameters for µ-CT-imaging.

Tube Voltage /kVp	Tube Current/µA	Noise Reduction (Frame Averaging)	Rotation Steps/°	Projections	Isotropic Voxel Sidel Length/µm
65	123	4-fold	0.23	1440	8.93

**Table 2:** Parameters for ToF-SIMS images (Figure 6 and Figure 7) obtained in positive ion mode with M6 Hybrid SIMS (IONTOF GmbH, Münster, Germany).

Figure 6 and Figure 7	Empty Defect	PPGC + S 5:5	PPGC + S 3:7
Analysis Options			
Cycle Time	85 µs	85 µs	85 µs
Raster Mode	sawtooth	sawtooth	sawtooth
Primary Ion Current	0.6 pA	0.4 pA	0.3 pA
Pixel density	300 pixel/mm	250 pixel/mm	600 pixel/mm
Frame per Patch	3	3	1
Patch size	0.4 mm	0.4 mm	0.5 mm
Primary Ion Shots/Frame/Pixel	3	3	3
Number of Scans	3	3	6

**Table 3:** Parameters for measurements with IONTOF’s M6 Hybrid SIMS.

***Primary Beam***		
Species		Ar_8000_^+^
Energy	/eV	20,000
Current	/pA	100
FoV	/µm²	400 × 400
Total Dose		5 × 10^11^
Dose Density	/1/cm²	3 × 10^14^
Raster Mode		sawtooth
Micro Raster Size	/pixel	72 by 72
***Analysis options***		
Polarity		positive
Cycle Time	/µs	400
Mode		Orbitrap Depth profile
Cratersize	/µm²	567.1 × 567.1
Injection Time	/ms	2950
Duration	/s	2.6
Estimated Depth per Frame	/nm	1.02
Mass Resolution m/Δm (FWHM)	at m/z 70.07 (C_4_H_8_N^+^)	>425,000
Mass Range	/m/z	50–750
Number of Scans		400 scans

## Supplementary Materials

The following are available online at, Table S1: ToF-SIMS analysis with Bi^3+^ primary ions as analysis species, performed in positive ion mode, enables detection of mass signals for HAP, collagen, and strontium in rat bone cross-sections of empty defect group as well as groups containing PPGC + S 5:5 and PPGC + S 3:7 as biomaterial. Mass signals of mineralized bone tissue, derived from hydroxyapatite Ca_10_(PO_4_)_6_(OH)_2_, were assigned according to previous literature [24,45]. Fragments of collagen (amino acids glycine, proline, and hydroxyproline, main components of collagen type 1) were based on collagen peaks known from previous studies [24,45,46]. Mass signals for strontium were based on mass peaks known from the literature [24]. Figure S1: ToF-SIMS mass images of rat bone sections with an empty defect (a–f) compared to bone sections containing PPGC + S 5:5 (g–l) and PPGC + S 3:7 (m–r) as implanted biomaterials. (a), (g), (m) For each group mass signals of mineralized bone in form of hydroxyapatite (HAP, signals are listed in Table S1) show the mass distribution of HAP in cortical and trabecular bone. (b), (h), (n) Collagen mass images show collagen structure in former defect area, cortical and trabecular bone, as well as bone marrow area. (c), (i), (o) Mass images of strontium signals (listed in Table S1) show natural, low-intensity occurrence of strontium in the empty defect. In comparison, strontium distribution in bone sections containing biomaterial is shown in remaining biomaterial fragments (i) as well as in lower intensity in cortical and trabecular bone. (d), (j), (p) RGB overlay images show mass fragments of mineralized bone in form of HAP (hydroxyapatite, red), non-mineralized collagen (green), and strontium signals (blue) (respective mass signals listed in Table S1). (e), (k), (q) Mass images of signals related to cartilage (a combination of sulfate signals Na_3_SO_4_^+^ and Na_5_S_2_O_8_^+^) are shown. (f), (l), (r) RGB overlay images show the mass distribution of HAP (mineralized bone, red), collagen mass signals (green) (listed in Table S1), as well as signals related to cartilage in blue. 25 keV Bi^3+^ primary ions were used for ToF-SIMS imaging in positive ion mode. (Scale bars = 2 mm). Figure S2: ToF-SIMS mass images and mass spectra of rat bone sections with an empty defect compared to bone sections containing PPGC + S 5:5 and PPGC + S 3:7 as implanted biomaterials. RGB overlay images show mass fragments of mineralized bone in form of HAP (hydroxyapatite) in red, non-mineralized collagen in green, and strontium signals in blue (respective mass signals listed in Table S1). For spectrometric measurements, Q Exactive™ orbital trapping mass spectrometer was used with 20 keV Ar_8000_^+^ clusters as primary ion species. Mass spectra were measured on different ROIs of one bone section of each group (1-9, ROIs are indicated by white rectangles). Sr^+^ signals were detected in areas of remaining biomaterials PPGC + S 5:5 (d) and PPGC + S 3:7 (g). In non-mineralized bone tissue, no strontium was detected. The white hollow arrows indicate remaining biomaterial fragments, white asterisks show former places of screws. Figure S3: Highest ED1 activity seen in the PPGC + S 5:5 group compared to PPGC + S 3:7 and empty defect, respectively. Table S2: Primer sequences and amplicon length for the different target genes.
