# Supplementary material for: Gelatin-Modified Calcium/Strontium Hydrogen Phosphates Stimulate Bone Regeneration in Osteoblast/Osteoclast Co-Culture and in Osteoporotic Rat Femur Defects—In Vitro to In Vivo Translation
Source: Molecules. 2020 Nov 3;25(21):5103. doi: 10.3390/molecules25215103 (PMC7662833; doi:10.3390/molecules25215103)
Supplement: Supplementary file 1 [file molecules-25-05103-s001.pdf]

Article

Gelatin Modified Calcium/Strontium Hydrogen Phosphates Stimulate Bone Regeneration in Osteoblast/Osteoclast co-Culture and in Osteoporotic Rat Femur Defects – *in vitro* to *in vivo* Translation

Benjamin Kruppke [1,#,*], Seemun Ray [2,#], Volker Alt [2#], Marcus Rohnke [3], Christine Kern [3], Marian Kampschulte [4], Christiane Heinemann [1#], Matthäus Budak [2], Josephine Adam [1], Nils Döhner [2,3], Lucretia Franz-Forsthoffer [2], Thaqif El Khassawna [2], Christian Heiss [2,5], Thomas Hanke [1] and Uli Thormann [2,5]

[1] Technische Universität Dresden, Institute of Materials Science, Max Bergmann Center of Biomaterials, 01069 Dresden, Germany; Benjamin.Kruppke@tu-dresden.de, Christiane.Heinemann@tu-dresden.de, Josephine.Adam@tu-dresden.de, Thomas.Hanke@tu-dresden.de

[2] University Hospital Giessen-Marburg GmbH, Campus Giessen, Department of Trauma, Hand and Reconstructive Surgery, Rudolf-Buchheim-Str. 7, 35385 Giessen, Germany; Ulrich.Thormann@chiru.med.uni-giessen.de, Matthaeus.Budak@chiru.med.uni-giessen.de, Lucretia.Franz-Forsthoffer@med.uni-giessen.de, Thaqif.ElKhassawna@chiru.med.uni-giessen.de, Christian.Heiss@chiru.med.uni-giessen.de

[3] Justus-Liebig-Universität Giessen, Institute of Physical Chemistry, Heinrich-Buff-Ring 17, 35392 Gießen, Germany; Marcus.Rohnke@phys.chemie.uni-giessen.de, Christine.Kern@phys.chemie.uni-giessen.de, Nils.Doehner@med.uni-giessen.de

[4] Justus-Liebig-Universität Giessen, Klinik für Diagnostische und Interventionelle Radiologie, Klinikstr. 33, 35392 Gießen, Germany; Marian.Kampschulte@radiol.med.uni-giessen.de

[5] University Hospital Giessen-Marburg GmbH, Campus Giessen, Experimental Trauma Surgery, Rudolf-Buchheim-Str. 7, 35385 Giessen, Germany; Ulrich.Thormann@chiru.med.uni-giessen.de, Christian.Heiss@chiru.med.uni-giessen.de

[#] These authors have equal main contribution to the manuscript.

* Correspondence: Benjamin.Kruppke@tu-dresden.de; Tel.: +49 351 463 42762 (B.K.)

Supplementals

Table S1. ToF-SIMS analysis with Bi^{3+} primary ions as analysis species, performed in positive ion mode, enables detection of mass signals for HAP, collagen, and strontium in rat bone cross sections of empty defect group as well as groups containing PPGC+S 5:5 and PPGC+S 3:7 as biomaterial. Mass signals of mineralized bone tissue, derived from hydroxyapatite $Ca_{10}(PO_4)_6(OH)_2$, were assigned according to previous literature [24,44]. Fragments of collagen (amino acids glycine, proline and hydroxyproline, main components of collagen type 1) were based on collagen peaks known from previous studies [24,44,45]. Mass signals for strontium were based on mass peaks known from literature [24].

Mineralized bone signals (HAP)				Collagen signals		Strontium signals	
m/z	Peak label	*m/z*	Peak label	*m/z*	Peak label	*m/z*	Peak label
39.96	Ca^+	214.79	$Ca_3PO_4^+$	30.04	CH_4N^+	85.89	$^{86}Sr^+$
55.97	CaO^+	230.78	$Ca_3PO_5^+$	44.05	$C_2H_6N^+$	86.91	$^{87}Sr^+$
56.96	$CaOH^+$	286.71	$Ca_4PO_6^+$	70.06	$C_4H_8N^+$	87.89	Sr^+
95.92	Ca_2O^+			86.06	$C_4H_8NO^+$	88.90	SrH^+
102.92	$CaPO_2^+$					103.90	SrO^+
111.90	$Ca_2O_2^+$					104.90	$SrOH^+$
112.91	$Ca_2O_2H^+$					150.85	$SrPO_2^+$
118.92	$CaPO_3^+$					166.85	$SrPO_3^+$
134.88	$CaPO_4^+$					182.86	$SrPO_4^+$
151.84	$Ca_3O_2^+$					207.79	$Sr_2O_2^+$
158.85	$Ca_2PO_3^+$					208.82	$Sr_2O_2H^+$
168.84	$Ca_3O_3H^+$					254.80	$Sr_2PO_3^+$
174.84	$Ca_2PO_4^+$					270.76	$Sr_2PO_4^+$

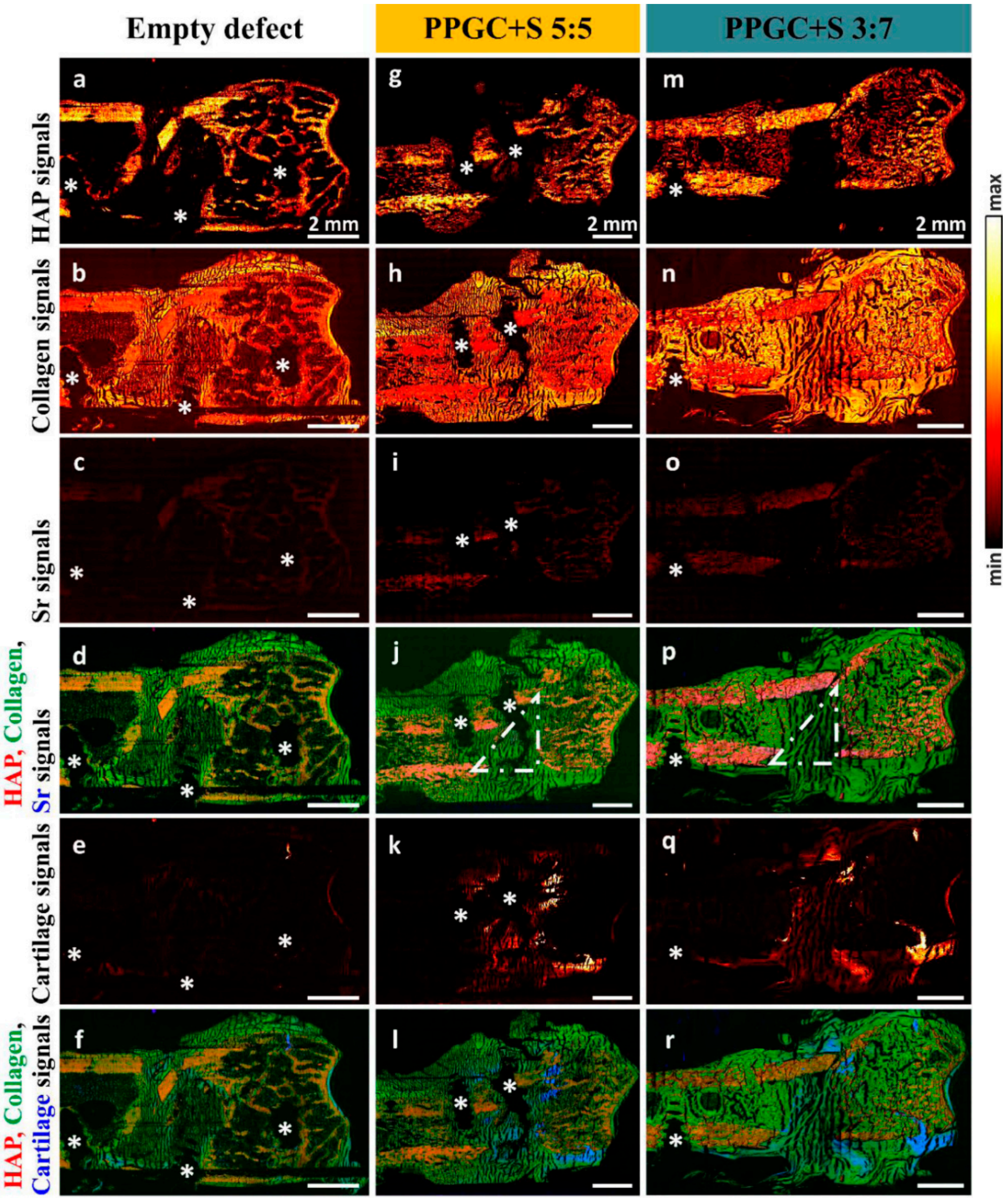

Figure S1. ToF-SIMS mass images of rat bone sections with an empty defect (**a-f**) compared to bone sections containing PPGC+S 5:5 (**g-l**) and PPGC+S 3:7 (**m-r**) as implanted biomaterials. (**a**), (**g**), (**m**) For each group mass signals of mineralized bone in form of hydroxyapatite (HAP, signals are listed in **Table S1**) show mass distribution of HAP in cortical and trabecular bone. (**b**), (**h**), (**n**) Collagen mass images show collagen structure in former defect area, cortical and trabecular bone as well as bone marrow area. (**c**), (**i**), (**o**) Mass images of strontium signals (listed in **Table S1**) show natural, low intensity occurrence of strontium in empty defect. In comparison, strontium distribution in bone sections containing biomaterial is shown in remaining biomaterial fragments (**i**) as well as in lower intensity in cortical and trabecular bone. (**d**), (**j**), (**p**) RGB overlay images show mass fragments of mineralized bone in form of HAP (hydroxyapatite, red), non-mineralized collagen (green), and strontium signals (blue) (respective mass signals listed in **Table S1**). (**e**), (**k**), (**q**) Mass

images of signals related to cartilage (combination of sulfate signals $Na_3SO_4^+$ and $Na_5S_2O_8^+$) are shown. (**f**), (**l**), (**r**) RGB overlay images show mass distribution of HAP (mineralized bone, red), collagen mass signals (green) (listed in **Table S1**) as well as signals related to cartilage in blue. 25 keV Bi_{3+} primary ions were used for ToF-SIMS imaging in positive ion mode. (Scale bars=2 mm)

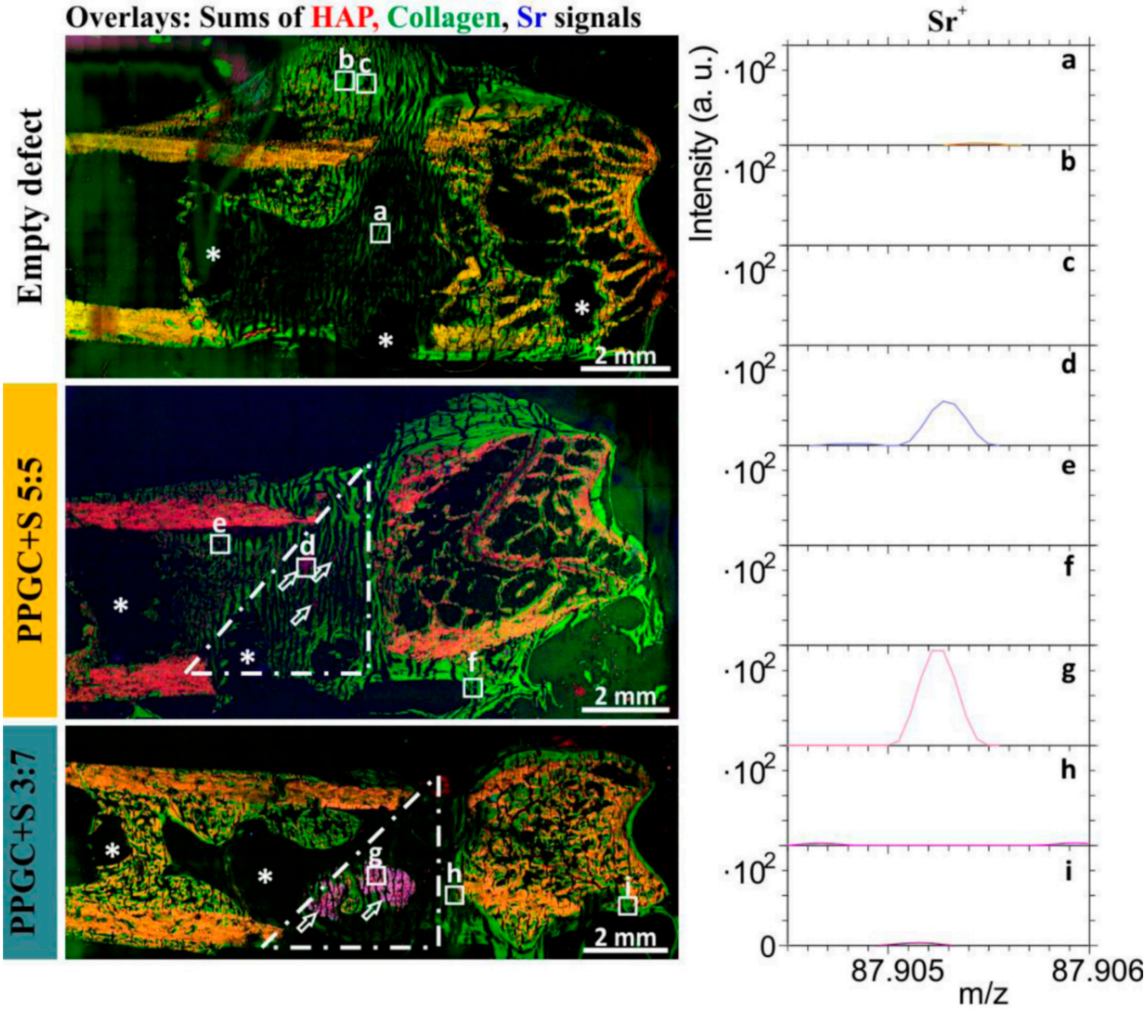

Figure S2. ToF-SIMS mass images and mass spectra of rat bone sections with an empty defect compared to bone sections containing PPGC+S 5:5 and PPGC+S 3:7 as implanted biomaterials. RGB overlay images show mass fragments of mineralized bone in form of HAP (hydroxyapatite) in red, non-mineralized collagen in green, and strontium signals in blue (respective mass signals listed in **Table S1**). For spectrometric measurements, Q Exactive™ orbital trapping mass spectrometer was used with 20 keV Ar_{8000}^+ clusters as primary ion species. Mass spectra were measured on different ROIs of one bone section of each group (1-9, ROIs are indicated by white rectangles). Sr^+ signals were detected in areas of remaining biomaterials PPGC+S 5:5 (**d**) and PPGC+S 3:7 (**g**). In non-mineralized bone tissue, no strontium was detected. The white hollow arrows indicate remaining biomaterial fragments, white asterisks show former places of screws.

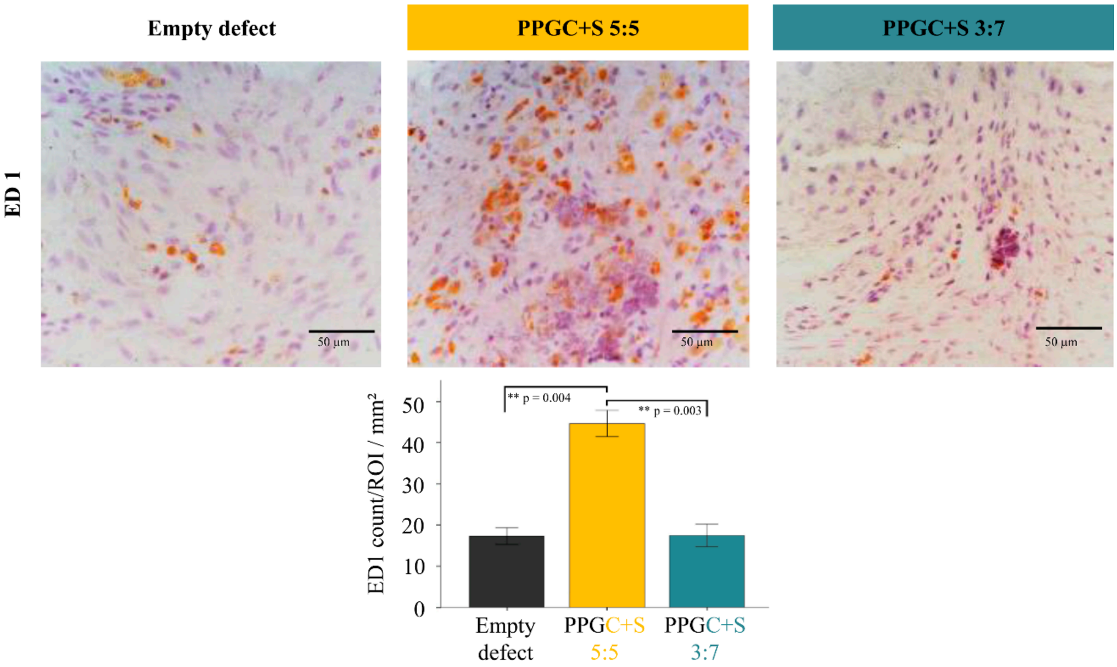

Figure S3 : Highest ED1 activity seen in the PPGC+S 5:5 group compared to PPGC+S 3:7 and empty defect respectively.

Table S2: Primer sequences and amplicon length for the different target genes.

Target Gene	Sense, antisense primers (5'-3') Sequence	Amplicon length (bp)
**B2M**	TGT CTC AGT TCC ACC CAC CT<br>GGG CTC CTT CAG AGT GAC G	191
**OCN**	GAG GGC AGT AAG GTG GTG AA<br>GTC CGC TAG CTC GTC ACA AT	135
**ALPL**	ATC GGA CCC TGC CTT ACC<br>CTC TTG GGC TTG CTG TCG	78
**Runx2**	CCA TAA CGG TCT TCA CAA ATC C<br>GCG GTC AGA GAA CAA ACT AGG	137
**Col1a1**	TCC TGA CGC ATG GCC AAG AA<br>CAT AGC ACG CCA TCG CAC AC	145
**Col10a1**	CAT GTG AAG GGG ACT CAC G<br>GAA GCC TGA TCC AAG TAG CC	101
**Car2**	GCC CCT GCT GGA ATG TGT GA<br>TGA GCT GGA CGC CAG TTG TC	144
**RANKL**	AAA TTA GCG TCC AGG TGT CC<br>TTG AAA GCC CCA AAG TAC G	73
**OPG**	ATG AAC AAG TGG CTG TGC TG<br>AAA GGT TTC CTG GGT TGT CC	72
